# High quality draft genome sequence and analysis of *Pontibacter roseus* type strain SRC-1^T^ (DSM 17521^T^) isolated from muddy waters of a drainage system in Chandigarh, India

**DOI:** 10.1186/1944-3277-10-8

**Published:** 2015-02-09

**Authors:** Supratim Mukherjee, Alla Lapidus, Nicole Shapiro, Jan-Fang Cheng, James Han, TBK Reddy, Marcel Huntemann, Natalia Ivanova, Natalia Mikhailova, Amy Chen, Krishna Palaniappan, Stefan Spring, Markus Göker, Victor Markowitz, Tanja Woyke, Brian J Tindall, Hans-Peter Klenk, Nikos C Kyrpides, Amrita Pati

**Affiliations:** 1 DOE Joint Genome Institute, Walnut Creek, California, USA; 2 T. Dobzhansky Center for Genome Bionformatics, St. Petersburg State University, St. Petersburg, Russia; 3 Algorithmic Biology Lab, St. Petersburg Academic University, St. Petersburg, Russia; 4 Leibniz Institute DSMZ – German Collection of Microorganisms and Cell Cultures, Braunschweig, Germany; 5 Biological Data Management and Technology Center, Lawrence Berkeley National Laboratory, Berkeley, California, USA; 6 King Abdulaziz University, Jeddah, Saudi Arabia

**Keywords:** Aerobic, Gram-negative, Non-motile, Obligate aerobe, Halotolerant, Menaquinone, GEBA, KMG-I

## Abstract

*Pontibacter roseus* is a member of genus *Pontibacter* family *Cytophagaceae*, class *Cytophagia*. While the type species of the genus *Pontibacter actiniarum* was isolated in 2005 from a marine environment, subsequent species of the same genus have been found in different types of habitats ranging from seawater, sediment, desert soil, rhizosphere, contaminated sites, solar saltern and muddy water. Here we describe the features of *Pontibacter roseus* strain SRC-1^T^ along with its complete genome sequence and annotation from a culture of DSM 17521^T^. The 4,581,480 bp long draft genome consists of 12 scaffolds with 4,003 protein-coding and 50 RNA genes and is a part of *Genomic Encyclopedia of Type Strains*: KMG-I project.

## Introduction

The genus *Pontibacter* was first reported by Nedashkovskaya *et al. *[1] where they identified and described a menaquinone producing strain isolated from sea anemones. Several new species of the same genus have been reported in the literature since then. In addition to *Pontibacter roseus*, there are eighteen species with validly published names belonging to *Pontibacter* genus as of writing this manuscript. Members of genus *Pontibacter* including *P. roseus*, is of interest for genomic research due to their ability to synthesize and use menaquinone-7 (MK-7) as the primary respiratory quinone as well as to facilitate functional genomics studies within the group. Strain SRC-1^T^ (= DSM 17521 = CCTCC AB 207222 = CIP 109903 = MTCC 7260) is the type strain of *Pontibacter roseus*, which was isolated from muddy water from an occasional drainage system of a residential area in Chandigarh, India [2]. *P. roseus* SRC-1^T^ was initially reported to be *Effluviibacter roseus* SRC-1^T^ primarily due to its non-motile nature and fatty acid composition [2]. However, subsequent analysis of its fatty acid profile was shown to be more ‘*Pontibacter*-like’ and gliding motility was observed to be variable in other *Pontibacter* species [3]. Further, its DNA G + C content, which was originally reported as 59 mol% [2], was also emended to be 52.0–52.3 mol% [3], a value more representative of members of the genus *Pontibacter*. As such, it was reclassified as *Pontibacter roseus* SRC-1^T ^[3]. Here we present a summary classification and features for *Pontibacter roseus* SRC-1^T^, along with the genome sequence and annotation of DSM 17521^T^.

## Organism information

### Classification and features

*P. roseus* SRC-1^T^ cells are non-motile, stain Gram-negative, do not form spores and are rod-shaped approximately 1.0–3.0 μm in length and 0.3–0.5 μm in width [2]. It is an obligate aerobe which can grow at a wide temperature range of 4–37°C with the optimum being 30°C (Table 1 and [2]). *P. roseus* SRC-1^T^ is a halotolerant microbe, can tolerate up to 8% NaCl and can utilize a wide range of sugars such as D-fructose, D-galactose, D-glucose, lactose, raffinose and sucrose as the sole source of carbon (Table 1 and [2]).

**Table 1:** Classification and general features of *Pontibacter roseus* SRC-1^T^ according to the MIGS recommendations [4], published by the Genomic Standards Consortium [5]

**MIGS ID**	**Property**	**Term**	**Evidence code**
		Domain *Bacteria*	TAS [6]
		Phylum *Bacteroidetes*	TAS [7,8]
		Class *Cytophagia*	TAS [8,9]
	Current classification	Order *Cytophagales*	TAS [10,11]
		Family *Cytophagaceae*	TAS [10,12]
		Genus *Pontibacter*	TAS [2,3,10]
		Species *Pontibacter roseus*	TAS [2,3,10]
	Strain	SRC-1^T^	TAS [2,3,10]
	Gram stain	Gram-negative	TAS [2]
	Cell shape	Irregular rods	TAS [2]
	Motility	Non-motile	TAS [2]
	Sporulation	Non-sporulating	TAS [2]
	Temperature range	4–37°C	TAS [2]
	Optimum temperature	30°C	TAS [2]
	Salinity	Halotolerant	TAS [2]
	Relationship to oxygen	Obligate aerobe	TAS [2]
	Carbon source	Sugars (Glucose, Galactose etc.)	TAS [2]
			TAS [2]
MIGS-6	Habitat	Wastewater, aquatic	TAS [2]
MIGS-6.2	pH	pH 6.0–10.0	TAS [2]
MIGS-15	Biotic relationship	Free living	NAS
MIGS-14	Known pathogenicity	Not reported	
	Biosafety level	1	NAS
MIGS-23	Isolation	Muddy water	TAS [2]
MIGS-4	Geographic location	Chandigarh, India	TAS [2]
MIGS-5	Time of sample collection	Before 2006	TAS [2]
MIGS-4.1	Latitude	30.733	TAS [2]
MIGS-4.2	Longitude	76.779	TAS [2]

Evidence codes – TAS: Traceable Author Statement (i.e., a direct report exists in the literature); NAS: Non-traceable Author Statement (i.e., not directly observed for the living, isolated sample, but based on a generally accepted property for the species, or anecdotal evidence); Evidence codes are from the Gene Ontology project [13].

**Figure 1:**
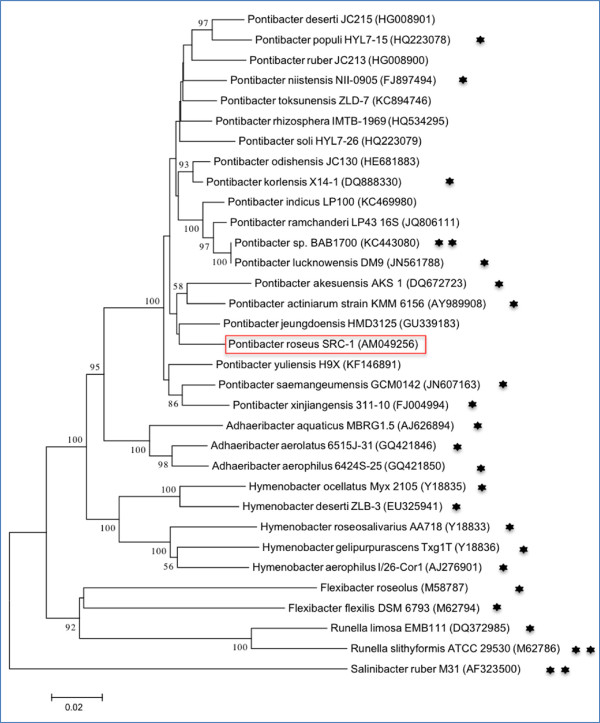
**Neighbour-joining phylogenetic tree based on 16S rRNA gene sequences, showing the relationships of *Pontibacter roseus*** SRC-1^T^**to other published *Pontibacter *type strains and representative type strains of the family *Cytophagace*ae with *Salinibacter ruber *M31 as the outgroup.** The neighbor joining [19] tree was constructed using MEGA v5.2.2 [20] based on the p-distance model with bootstrap values >50 (expressed as percentages of 1,000 replicates) shown at branch points. Lineages with type strain genome sequencing projects registered in GOLD [21] are labeled with one asterisk, while those with a published genome sequence is marked with two asterisks [16,22,23].

A representative genomic 16S rRNA sequence of *Pontibacter roseus* SRC-1^T^ was compared with the May 2013; release 13_5 of Greengenes database [14] using NCBI BLAST under default values. The top 250 hits with an alignment length cut-off of 1000 bp were retained among which genomes belonging to genus *Pontibacter* were the most abundant (45.6%) followed by *Adhaeribacter* (35.6%), those assigned to the family *Cytophagaceae* but without a defined genus name (16.4%) and *Hymenobacter* (2.4%). Among samples with available metadata, approximately 61% of the above hits were from a soil environment, 11% were isolated from skin and approximately 9% from aquatic samples. This distribution reflects the wide range of habitats commonly observed among members of the genus *Pontibacter* and its phylogenetic neighbors*,* ranging from forest soil to desert, contaminated aquatic and soil environments, sediments and seawater among others [15-18]. Figure 1 shows the phylogenetic neighborhood of *Pontibacter roseus* SRC-1^T^ in a 16S rRNA based tree.

The predominant respiratory quinone for strain SRC-1^T^ is menaquinone 7 (MK-7), consistent with other members of the *Pontibacter* genus. Short chain menaquinones with six or seven isoprene units are characteristic of the different genera within the aerobic members of the phylum *Bacteroidetes*. The primary whole-cell fatty acids are branched chain iso-C_15 : 0_ (14%), iso-C_17 : 0_ 3-OH (14.7%) and summed feature 4 (34.9%, comprising of anteiso-C_17 : 1_ B and/or iso-C_17 : 1_ I, a pair of fatty acids that are grouped together for the purpose of evaluation by the Microbial Identification System(MIDI) as described earlier [24]) [2,3]. 2-OH Fatty acids are absent. The original paper describing *P. roseus* SRC-1^T^ (as *Effluviibacter roseus*) [2] lists the polar lipids in strain SRC-1^T^ being phosphatidylglycerol, diphosphatidylglycerol and an unknown phospholipid. This is in stark contrast to the known lipid profile of this evolutionary group where phosphatidylethanolamine is usually the sole major digylceride based phospholipid and other non-phosphate based lipids make up a significant proportion of the polar lipids. Accordingly, while genes for phosphatidylserine synthase and a decarboxylase to convert the serine to phosphatidylethanolamine could be detected, we did not find any evidence in *P. roseus* DSM 17521^T^ genome to indicate that it produces the corresponding enzymes involved in the synthesis of phosphatidylglycerol or diphosphatidylglycerol. We therefore conclude that the original report on the lipid composition of strain SRC-1^T^ is probably in error. It should be noted that the original publication did not provide images of the TLC plates allowing others to examine these data set [2].

## Genome sequencing and annotation

### Genome project history

This organism was selected for sequencing on the basis of its phylogenetic position [25,26]. It is a part of the *Genomic Encyclopedia of Type Strains*, KMG-I project [27], a follow-up of the GEBA project [28], which aims to increase the sequencing coverage of key reference microbial genomes and to generate a large genomic basis for the discovery of genes encoding novel enzymes [29]. KMG-I is a Genomic Standards Consortium project [30]. The genome project is deposited in the Genomes OnLine Database [21], the annotated genome is publicly available from the IMG Database [31] under the accession 2515154084, and the permanent draft genome sequence has been deposited at GenBank under accession number ARDO00000000. Sequencing, finishing and annotation were performed by the DOE Joint Genome Institute (JGI) using state of the art technology [32]. The project information is briefly summarized in Table 2.

**Table 2:** Project information

**MIGS ID**	**Property**	**Term**
MIGS-31	Finishing quality	High-Quality draft
MIGS-28	Libraries used	Illumina Std shotgun library
MIGS-29	Sequencing platforms	Illumina HiSeq 2000
MIGS-31.2	Sequencing coverage	122.8 × Illumina
MIGS-30	Assemblers	Velvet v. 1.1.04, ALLPATHS v. R41043
MIGS-35	GC Content	52.65%
	INSDC ID	ARDO01000000
	GOLD ID	Gi11777
	NCBI project ID	169723
	Release date	08-13-2012
	Database: IMG	2515154084
MIGS-13	Source material identifier	DSM 17521
	Project relevance	GEBA-KMG, Tree of Life

### Growth conditions and DNA isolation

*Pontibacter roseus* DSM 17521^T^, was grown aerobically in DSMZ medium 948 (Oxoid nutrient broth) [33] at 30°C. Genomic DNA was isolated using a Jetflex Genomic DNA Purification Kit (GENOMED 600100) following the standard protocol provided by the manufacturer with the following modifications: an additional incubation (60 min, 37°C) with 50 μl proteinase K and finally adding 200 μl protein precipitation buffer (PPT). DNA is available through the DNA Bank Network [34].

### Genome sequencing and assembly

The draft genome of *Pontibacter roseus* DSM 17521^T^ was generated at the DOE-JGI using the Illumina technology [35]. An Illumina Std shotgun library was constructed and sequenced using the Illumina HiSeq 2000 platform which generated 12,071,874 reads totaling 1,810.8 Mbp. All general aspects of library construction and sequencing performed at the JGI is publicly available [36]. All raw Illumina sequence data was passed through DUK, a filtering program developed at JGI, which removes known Illumina sequencing and library preparation artifacts. Following steps were then performed for assembly: (1) filtered Illumina reads were assembled using Velvet (version 1.1.04) [37], (2) 1–3 Kbp simulated paired end reads were created from Velvet contigs using wgsim [38], (3) Illumina reads were assembled with simulated read pairs using Allpaths–LG (version r41043) [39]. Parameters for assembly steps were: 1) Velvet (velveth: 63 –shortPaired and velvetg: -very clean yes –export- Filtered yes –min_contig_lgth 500 –scaffolding no –cov_cutoff 10) 2) wgsim (-e 0 –1 100 –2 100 –r 0 –R 0 –X 0) 3) Allpaths–LG (PrepareAllpathsInputs: PHRED_64 = 1 PLOIDY = 1 FRAG_COVERAGE = 125 JUMP_COVERAGE = 25 LONG_JUMP_COV = 50, RunAllpathsLG: THREADS = 8 RUN = std_shredpairs TARGETS = standard VAPI_WARN_ONLY = True.

OVERWRITE = True). The final draft assembly contained 12 scaffolds. The total size of the genome is 4.6 Mbp and the final assembly is based on 562.0 Mbp of Illumina data, which provides an average 122.8 × coverage of the genome. Additional information about the organism and its genome sequence and their associated MIGS record is provided in Additional file 1.

### Genome annotation

Genes were identified using Prodigal [40] as part of the JGI genome annotation pipeline [41], followed by a round of manual curation using the JGI GenePRIMP pipeline [42]. The predicted CDSs were translated and used to search the NCBI nonredundant database, UniProt, TIGRFam, Pfam, PRIAM, KEGG, COG, and InterPro databases. These data sources were combined to assert a product description for each predicted protein. Non-coding genes and miscellaneous features were predicted using tRNAscan-SE [43], RNAMMer [44], Rfam [45], TMHMM [46], SignalP [47] and CRT [48]. Additional gene functional annotation and comparative analysis were performed within the IMG platform [49].

## Genome properties

**Figure 2:**
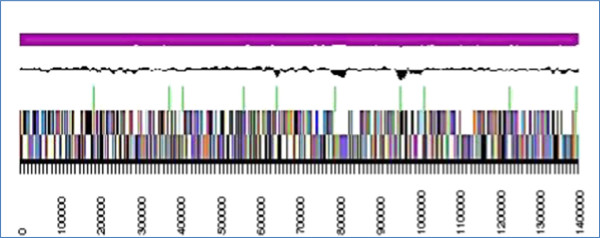
**The graphical map of the largest scaffold of the genome.** From bottom to the top: Genes on forward strand (color by COG categories), Genes on reverse strand (color by COG categories), RNA genes (tRNA green, rRNA red, other RNAs black), GC content, GC skew (purple/olive).

The assembly of the draft genome sequence consists of 12 scaffolds amounting to a 4,581,480 bp long chromosome with a GC content of approximately 53% (Table 3 and Figure 2). Of the 4,053 genes predicted, 4,003 were protein-coding genes along with 50 RNAs. The majority of protein-coding genes (69.4%) were assigned with a putative function while the remaining ones were annotated as hypothetical proteins.

**Table 3:** Genome statistics

**Attribute**	**Value**
Genome size (bp)	4,581,480
DNA coding (bp)	3,984,478
DNA G + C (bp)	2,411,942
Total genes	4,053
RNA genes	50
rRNA operons	1
tRNA genes	41
Protein-coding genes	4,003
Pseudo genes	-
Genes with function prediction	2,813
Genes in paralog clusters	1,373
Genes assigned to COGs	2,790
Genes assigned Pfam domains	3,062
Genes with signal peptides	611
Genes with transmembrane helices	997

The functional distribution of genes assigned to COGs is shown in Table 4. A large percentage of the genes do not have an assigned COG category, are unknown or fall into general function prediction, which is typical for a newly sequenced organism that has not been well characterized yet.

**Table 4:** Number of genes associated with the 25 general COG functional categories

**Code**	**Value**	**% of total**^**a**^	**Description**
J	167	4.17	Translation
A	1	0.02	RNA processing and modification
K	134	3.35	Transcription
L	105	2.62	Replication, recombination and repair
B	1	0.02	Chromatin structure and dynamics
D	27	0.67	Cell cycle control, mitosis and meiosis
Y	0	0.00	Nuclear structure
V	60	1.50	Defense mechanisms
T	94	2.35	Signal transduction mechanisms
M	229	5.72	Cell wall/membrane biogenesis
N	11	0.27	Cell motility
Z	0	0.00	Cytoskeleton
W	0	0.00	Extracellular structures
U	30	0.75	Intracellular trafficking and secretion
O	94	2.35	Posttranslational modification, protein turnover, chaperones
C	153	3.82	Energy production and conversion
G	126	3.15	Carbohydrate transport and metabolism
E	203	5.07	Amino acid transport and metabolism
F	65	1.62	Nucleotide transport and metabolism
H	99	2.47	Coenzyme transport and metabolism
I	96	2.40	Lipid transport and metabolism
P	149	3.72	Inorganic ion transport and metabolism
Q	69	1.72	Secondary metabolites biosynthesis, transport and catabolism
R	328	8.19	General function prediction only
S	229	5.72	Function unknown
-	1790	44.72	Not in COGs

^a^The total is based on the total number of protein coding genes in the annotated genome.

## Insights from the genome sequence

### Menaquinone biosynthesis

Respiratory lipoquinones such as ubiquinone and menaquinone are essential components of the electron transfer pathway in bacteria and archaea. While ubiquinones are limited to members of *Alphaproteobacteria*, *Gammaproteobacteria* and *Betaproteobacteria* [50], menaquinones have been found to be more widespread among prokaryotes [51,52], occurring in both aerobes and anaerobes. Menaquinone is a non-protein lipid-soluble redox component of the electron transport chain, which plays an important role in mediating electron transfer between membrane-bound protein complexes. The classical menaquinone biosynthesis pathway was studied primarily in *Escherichia coli*; more recently, an alternate pathway was identified in *Streptomyces coelicolor* A3(2) as well as in pathogens such as *Helicobacter pylori* and *Campylobacter jejuni* [53,54], aspects of which remain to be fully elucidated.

All identified species of the genus *Pontibacter* are known to possess menaquinone – 7 [16] which is the primary respiratory quinone in *Pontibacter roseus* SRC-1^T ^[2]. Biosynthesis of menaquinone in this organism appears to occur via the classical pathway. Using comparative genomics we identified the genes possibly involved in menaquinone biosynthesis in *P. roseus* DSM 17521^T^ (Table 5). Menaquinone biosynthesis genes have been extensively studied in *E. coli* where they are organized in an operon and in *B. subtilis* where gene neighborhood was helpful in identifying *men*C and *men*H genes [61]. However, the *P. roseus* genes seem to be spread across its chromosome. It is well known that conservation of gene order in bacteria can be disrupted during the course of evolution [62]. For example, isolated genes belonging to the menaquinone biosynthesis pathway leading to phylloquinione biosynthesis were identified in *Synechocystis* sp. PCC 6803 through sequence similarity with *E. coli* followed by transposon mutagenesis [63,64]. As more genomes become available, these aspects can be investigated in greater detail.

**Table 5:** **Predicted menaquinone biosynthesis genes in *Pontibacter roseus *** **DSM 17521**^**T**^

**IMG GeneID**	**IMG description**	**Identity to characterized proteins**	**Reference**
2515478196	isochorismate synthases	28% identity to *E. coli* MenF	[55]
2515478195	2-succinyl-5-enolpyruvyl-6-hydroxy-3-cyclohexene-1-carboxylic-acid synthase	30% identity to *B. subtilis* MenD	[56]
2515478193	Acyl-CoA synthetases	26% identity to *E. coli* MenE	[57]
2515478204	naphthoate synthase	52% identity to *E. coli* MenB	[58]
2515479036	1,4-dihydroxy-2-naphthoate octaprenyltransferase	31% identity to *E. coli* MenA	[59]
2515480441	muconate and chloromuconate cycloisomerases	48% identity with muconate cycloisomerase 1 of *Pseudomonas putida*	[60]

**Figure 3:**
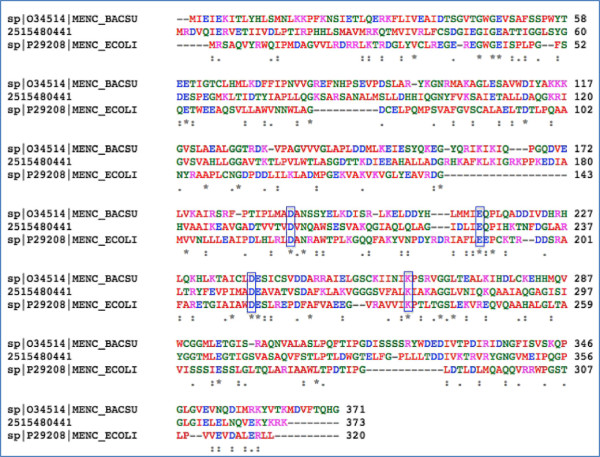
**Multiple sequence alignment of MenC from *E. coli*, *B. subtilis *** **and predicted MenC in *P. roseus *** **(IMG 2515480441) showing conserved amino acids predicted to be essential for proper functioning of the enzyme.**

An o-succinylbenzoate synthase that is part of the menaquinone biosynthetic pathway encoded by the *men*C gene in *E. coli* and *B. subtilis* is missing from the *Pontibacter roseus* genome. A gene annotated as muconate cycloisomerase in *Pontibacter roseus* DSM 17521^T^ (IMG geneID 2515480441) may perform this function. It contains conserved domains belonging to Muconate Lactonizing Enzyme subgroup of the enolase superfamily. Sequence similarity between different members of the enolase superfamily is typically less than 25% [65]. Even though they possess similar structural scaffolds, they are known to have evolved significantly such that their functional role cannot be easily assigned through sequence similarity alone [66]. For example, *B. subtilis men*C was initially annotated as ‘similar to muconate cycloisomerase of *Pseudomonas putida*’ and ‘N-acylamino acid racemase’ but was later corrected to be OSBS [61]. The *P. roseus* gene shares protein level identity of 48% with muconate cycloisomerase 1 of *Pseudomonas putida* [60] and approximately 23% and 17% with *E. coli* and *B. subtilis* MenC respectively [61,67]. Multiple sequence alignment (Figure 3) of the above three genes reveal conservation of Asp^161^, Glu^190^, Asp^213^ and Lys^235^ (boxes in Figure 3) which have been predicted to be essential for OSBS in *E. coli* and other members of the enzyme family [65]. We thereby propose that IMG 2515480441 performs the function of MenC in *P. roseus* DSM 17521^T^.

### Multidrug resistance (MDR) efflux pump

**Figure 4:**
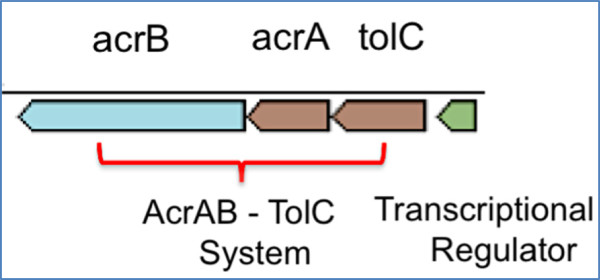
**Predicted MDR efflux system in *P. roseus *** **with AcrAB-TolC along with a transcriptional regulator.**

Resistance to antibiotic drugs is one of the major public health concerns of today as highlighted in the recent report by the CDC [68]. Among several other mechanisms, multidrug resistance efflux pumps play a very important role in conferring decreased susceptibility to antibiotics in bacteria by transporting drugs across the bacterial membrane and preventing intracellular accumulation [69]. AcrAB-TolC is one of the most studied MDR efflux systems in Gram-negative bacteria. It is comprised of an inner membrane efflux transporter (AcrB), a linker protein (AcrA) and an outer membrane protein (TolC), which interacts with AcrA and AcrB and forms a multifunctional channel that is essential to pump cellular products out of the cell [69,70]. Previous reports have identified gene clusters predicted to confer antibiotic resistance in members of *Pontibacter* [16]. Applying comparative analysis with characterized proteins, we identified a set of genes (IMG ID 2515478940–43) that may function as a multi-drug resistance efflux pump in *P. roseus* DSM 17521^T^ (Figure 4).

*P. roseus* 2515478940 is 37% identical to *E. coli* multidrug efflux pump subunit AcrB [71]; 2515478941 shares 28% identity to *E. coli* AcrA [72] while 2515478942 is 20% identical to *E. coli* outer membrane protein TolC [73]. Additionally, there is a transcriptional repressor (2515478943) upstream of TolC which shares 25% protein level identity to HTH-type transcriptional repressor Bm3R1 [74] from *Bacillus megaterium* and may act as a regulator of the MDR transport system in *P. roseus* DSM 17521^T^.

## Conclusions

Members of the genus *Pontibacter* occupy a unique phylogenetic niche within the phylum *Bacteroidetes*. As of writing, this genome report is only the second for the entire genus. In addition to a detailed analysis of the *P. roseus* genome we highlight some of the key functional characteristics of the organism and summarize the genes encoding enzymes leading to the biosynthesis of menaquinone, the primary respiratory quinone for majority of the species of the genus.

## Abbreviations

KMG: One thousand microbial genomes; OSBS: o-succinylbenzoate synthase; MDR: Multi-Drug Resistance.

## Competing interests

The authors declare that they have no competing interests.

## Authors’ contributions

SM, BJT, SS, MG, HPK, NCK and AP drafted the manuscript. AL, NS, J-FC, JH, TBKR, MH, NI, NM, AC, KP and TW sequenced, assembled and annotated the genome. All authors read and approved the final manuscript.

## Supplementary Material

Additional file 1 Associated MIGS record. Click here for file
