# Supplementary material for: High quality draft genome sequence and analysis of Pontibacter roseus type strain SRC-1T (DSM 17521T) isolated from muddy waters of a drainage system in Chandigarh, India
Source: Stand Genomic Sci. 2015 Feb 9;10:8. doi: 10.1186/1944-3277-10-8 (PMC4511580; doi:10.1186/1944-3277-10-8)
Supplement: Additional file 1 — Associated MIGS record. [file 1944-3277-10-8-S1.docx]

**Table S1.** Associated MIGS record

| **MIGS-ID** | field name | description |
| --- | --- | --- |
| **MIGS-1** | Submit to INSDC/Trace archives | INSDC ID ARDO01000000 |
| **MIGS-2** | MIGS CHECK LIST TYPE |  |
| **MIGS-3** | Current classification  Project Name | Domain Bacteria  Phylum Bacteroidetes  Class Cytophagia  Order Cytophagales  Family Cytophagaceae  Genus Pontibacter  Species roseus  Genomic Encyclopedia of Type Strains, Phase I: One thousand microbial genomes (KMG-I) |
| **MIGS-4** | Geographic Location |  |
| **4.1** | Latitude | 30.7333 |
| **4.2** | Longitude | 76.779 |
| **4.3** | Depth |  |
| **4.4** | Altitude |  |
| **MIGS-5** | Time of Sample collection | Before 2006 |
| **MIGS-6** | Habitat (EnvO) |  |
| **6.1** | temperature | 30°C |
| **6.2** | pH | 6.0 – 10.0 |
| **6.3** | salinity | Halotolerant |
| **6.4** | chlorophyll |  |
| **6.5** | conductivity |  |
|  |  |  |
| **6.6** | light intensity |  |
| **6.7** | dissolved organic carbon (DOC) |  |
| **6.8** | current |  |
| **6.9** | atmospheric data |  |
| **6.10** | density |  |
| **6.11** | alkalinity |  |
| **6.12** | dissolved oxygen |  |
| **6.13** | particulate organic carbon (POC) |  |
| **6.14** | phosphate |  |
| **6.15** | nitrate |  |
| **6.16** | sulfates |  |
| **6.17** | sulfides |  |
| **6.18** | primary production |  |
| **MIGS-7** | Subspecific genetic lineage | Strain SRC-1^T^ |
| **MIGS-9** | Number of replicons | 1 |
| **MIGS-10** | Extrachromosomal elements | 0 |
| **MIGS-11** | Estimated Size | 4581kb |
| **MIGS-12** | Reference for biomaterial or Genome report |  |
| **MIGS-13** | Source material identifiers | DSM17521 |
| **MIGS-14** | Known Pathogenicity | Not reported |
|  |  |  |
| **MIGS-15** | Biotic Relationship |  |
| **MIGS-16** | Specific Host |  |
| **MIGS-17** | Host specificity or range (taxid) |  |
| **MIGS-18** | Health status of Host |  |
| **MIGS-19** | Trophic Level | Chemeheterotroph |
| **MIGS-22** | Relationship to Oxygen | Obligate aerobe |
| **MIGS-23** | Isolation and Growth conditions | Muddy waters, DSMZ 948 |
| **MIGS-27** | Nucleic acid preparation | Jetflex DNA purification kit with modifications |
| **MIGS-28** | Library construction |  |
| **28.1** | Library size | 1810.8Mbp |
| **28.2** | Number of reads | 12,071,874 |
| **28.3** | vector |  |
| **MIGS-29** | Sequencing method | Illumina HiSeq 2000 |
| **MIGS-30** | Assembly |  |
| **30.1** | Assembly method |  |
| **30.2** | estimated error rate | Velvet v. 1.1.04, ALLPATHS v. R41043 |
| **30.3** | method of calculation |  |
| **MIGS-31** | Finishing strategy |  |
| **31.1** | Status | High-Quality draft |
| **31.2** | coverage | 122.8X Illumina |
| **31.3** | contigs | 15 |
| **MIGS-32** | Relevant SOPs | GOLD ID: Gi11777  IMG ID: 2515154084 |
| **MIGS-33** | Relevant e-resources |  |
